# Decision-making process in seeking antenatal care: A cohort study in a poor urban and a typical rural area in Bangladesh

**DOI:** 10.7189/jogh.14.04097

**Published:** 2024-05-17

**Authors:** Shakil Ahmed, Tanjeena Tahrin Islam, Fauzia Akhter Huda, Anadil Alam, Rashida Akter, Qazi Sadeq-ur Rahman, Quamrun Nahar, Shams El Arifeen, Mahbub Elahi Chowdhury

**Affiliations:** 1 Maternal and Child Health Division, icddr,b, Dhaka, Bangladesh; 2 Health Systems and Population Studies Division, icddr,b, Dhaka, Bangladesh

## Abstract

**Background:**

Decision-making in choosing and using maternal health care among different care-seeking options is a complex process influenced by multilevel factors. Existing evidence on maternal health care-seeking behaviour stems primarily from cross-sectional studies with limited information. Therefore, we designed a cohort study to better understand the decision-making process in antenatal care (ANC) seeking.

**Methods:**

We conducted this mixed-methods study among pregnant women at <27 weeks of gestation in a poor urban area (n = 1320) and a typical rural area of Bangladesh (n = 1239) whom we followed up till eight weeks after delivery. In view of quantitative methods, we interviewed all enrolled women 5–6 times four weeks apart. For the qualitative approach, we conducted 70 case studies in the urban area and 46 in the rural area by interviewing the participants and their close family members.

**Results:**

In the urban area, about one-third of the pregnant women (38.4%) sought ANC at non-governmental organisations, and nearly an equal proportion went to public facilities (36.6%). In both the situations, women preferred facilities with one-stop services at a reasonable cost. In contrast, the lack of readiness in public facilities of the rural area pushed women (77.8%) toward private facilities for ANC. The reputation of the facilities, availability of skilled care providers, diagnostic tests, and ultrasonography services therein were the key influencing factors in the participants’ decisions to seek ANC services from specific facilities.

**Conclusions:**

The availability of one-stop services was a key factor for participants’ choosing of a facility for ANC. For the urban setting, there is a need to establish large public facilities with one-stop service provision in different zones, along with supporting non-governmental organisations in poor areas. For the rural setting, there is an urgent need to strengthen ANC service provision in public facilities at the community- and the sub-district level to redirect women from the private to the public sector to ensure low cost, quality services.

Maternal and child health has been considered the topmost priority in Bangladesh’s national health strategy for the last three decades [1,2]. The ‘Three Delays’ framework, aimed at improving mother and child health indicators nationwide, has been crucial in shaping the country’s health policies. This framework specifically addresses delays in decision-making for seeking health care services, accessing health care facilities, and obtaining timely and quality care from facilities and care providers [1,3]. The establishment of the emergency obstetric care services in the national health system was a great step toward achieving the Millennium Development Goal for reducing maternal mortality in Bangladesh, but has not been sufficient for achieving the related target [4–6]. Multiple gaps and challenges still persist in the current era of Sustainable Development Goals, necessitating attention and concerted efforts in maternal and child health to reach the targets successfully [7].

Evidence has shown that antenatal care (ANC) is vital for safeguarding the health of pregnant women and their unborn children [8–10], as it promotes facility-based delivery which enables convenient access to emergency obstetric care services in the event of complications during childbirth [11]. On a global scale, it has been observed that 90% of pregnant women undergo at least one ANC visit to qualified health care practitioners. However, the number of women attending four ANC visits remains notably low [12]. Moreover, research has clearly indicated that regions with low rates of ANC-use are associated with high rates of maternal mortality [12–14]. According to the Bangladesh Demographic and Health Survey (BDHS) 2022, a significant proportion of pregnant women (88%) sought ANC from skilled health care providers, but only 40% undertook the four or more recommended ANC visits [15].

Similarly, while the percentage of pregnant women having at least one ANC visit in other low and middle-income countries was high, the likelihood of these women returning to the health care facility for four or more ANC visits remained low [16,17]. The decision-making process pertaining to the seeking and receiving of health care, particularly ANC, is a complex process influenced by multiple factors operating at different levels. These factors, however, often diverge from preferences and intentions of pregnant women [18,19]. Several cross-sectional studies have indicated that certain socioeconomic factors are associated with having less ANC, where families with lower income, lower levels of formal education, younger ages at marriage, higher numbers of previous pregnancies, fewer ANC visits, and less media exposure have been shown to be more prone to delivering at home [20,21].

A comprehensive understanding of the decision-making processes in ANC is crucial in explaining health care-seeking and service utilisation to close the gaps in service uptake. Healthcare-seeking behaviours of pregnant women are affected by the influence of spouses, household members, relatives, neighbours, and the community network [22]. Healthcare quality and availability may affect the ways how individuals, families, and communities see their treatment and its value [23]. Along with obvious barriers to health care access, location, travel time, and financial concerns may also affect health care-seeking, ultimately delaying individuals’ decision to undergo treatment [22,24].

Existing evidence on maternal care-seeking behaviour mostly comes from cross-sectional studies which are limited to data from a single time point. Therefore, we designed and conducted this cohort study to explore the dynamics of the decision-making process and to identify the factors influencing decision-making around seeking ANC in Bangladesh.

## METHODS

### Study design

This was a prospective cohort study of pregnant women implemented in an urban and a rural setting. We applied a mixed methods approach with both quantitative and qualitative research components, along with facility observation. The urban setting was a poor area in the northwestern part of Dhaka, the capital city of Bangladesh, while the rural setting was the Baliakandi *upazila* (sub-district) of the Rajbari district, situated about 150 km southwest of Dhaka.

**Figure 1:**
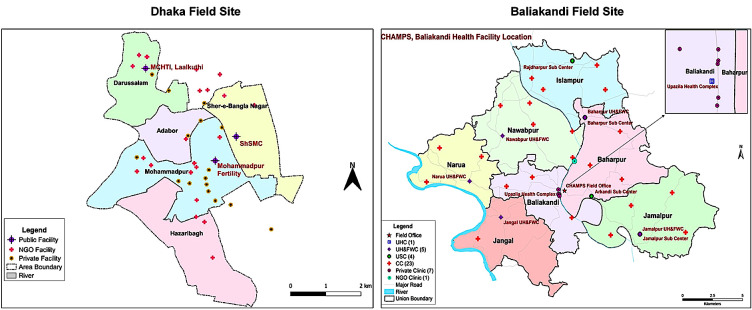
Map of study sites.

During our study, three major public facilities and more than 30 private facilities were serving the local population with maternity services in the vicinity of the urban setting (**Figure 1**). We identified the poor urban setting based on six important criteria: types of dwelling houses; population density; types of land; water supply; energy and infrastructure; and overall socioeconomic status of the dwellers [25].

Within the rural setting, we included all seven unions of the Baliakandi *Upazila*, where a health and demographic surveillance system of icddr,b was in operation, from which we selected our study samples. A 50-bedded *upazila* health complex (UHC), situated in the centre of the sub-district, was the main service delivery point in the public sector for the sub-district population. It was linked with the 100-bedded district hospital at the Rajbari district headquarters. Several private clinics (seven in Baliakandi and more than 20 in the Rajbari district centre) were actively serving clients from the study area for maternity care (**Figure 1**). Only one small non-governmental organisation (NGO) was functioning in this rural setting, and it was merged with the private facilities during data analysis.

We purposefully selected these two study areas to understand the dynamics of the participants’ decision-making process while choosing facilities and maternity services from a wide range of options. We collected data from September 2020 to December 2021 in the urban area and from December 2021 to November 2022 in the rural area.

### Study population and selection of pregnant women

We used two different approaches to enrol women from urban and rural areas, considering their feasibilities in different contexts. Specifically, we selected households with poor living facilities (in terms of house structure, population density, utility services, and road communication) in the urban area to carry out household listings using a checklist to identify the eligible pregnant women. Meanwhile, in the rural area, we collected the list of pregnant women from the health and demographic surveillance system of icddr,b, which included the general rural population. We only included women at less than 27 weeks of pregnancy confirmed by pregnancy test who had been living in the study area for at least six months. Thus, we enrolled 1522 and 1263 pregnant women from the urban and rural areas, respectively. We followed up the enrolled women in each setting at the household level every four weeks during the pregnancy period (3–4 times during pregnancy) and two times after delivery, first within 1–2 week(s) and then within 43–57 days after delivery.

### Data collection

#### Quantitative assessment

We interviewed the enrolled women at each follow-up visit for information relating to their health condition and care-seeking patterns during pregnancy, including factors relating to their decision-making in choosing the type of facility to be visited and the providers at different stages of their pregnancy. A trained female interviewer conducted all the interviews using a semi-structured questionnaire installed in tablet PCs. We followed up 1320 (attrition rate 13.2%) women in the urban and 1239 (attrition rate 1.9%) in the rural cohort until the fifty-seventh day of pregnancy outcomes.

#### Qualitative assessment

We conducted 70 case studies in the urban area and 46 in the rural area to explore participants’ decision-making process around ANC. Each case study was focussed on a pregnant women as the primary respondent, while the people who played important roles in her care-seeking process during the pregnancy period (generally mother-in-law, husband, and close relatives) were the secondary respondents for in-depth interviews.

We purposefully selected the qualitative samples based on the pregnant women’s care-seeking behaviour, such as changing facility for ANC visits at least once; not changing any facility throughout the whole pregnancy; or receiving ANC only at home. We also selected nine health facilities in the urban setting and six in the rural setting that were most frequently visited by the enrolled women for pregnancy care for observation in order to better understand how their service provisions played a role in the participants’ decision-making to seek ANC. In each of the selected facilities, two study team members documented the information on ANC service provision for 2–3 days, using facility observation guidelines and a checklist.

### Tools development and data-collection process

Trained female interviewers (12 for the urban site and eight for the rural site) collected data at the household level. One supervisor was assigned for every 4–6 interviewers. We developed their data collection tools through a consultative process. We finalised them after pre-testing and used them to develop an Android App installed in Android-based tablet PCs. Each tablet PC was linked via Internet to a computer server for synchronisation of the collected data to a password-secured server of icddr,b. To ensure data quality, we randomly selected 5% of the completed interviews and re-interviewed respondents with selected questions to validate the data. We then organised biweekly consultation meetings with the full team to discuss the findings from the validation and to clarify any issue that arose during field implementation. We also assigned one field manager for each site for the overall field implementation of the study, including monitoring quality of data collection. Furthermore, the qualitative in-depth interviews with pregnant women and their family members were performed using pre-tested guidelines, voice-recorded, and conducted by expert researchers with backgrounds in anthropology.

### Data analysis

We presented the outcomes using descriptive statistics, performed z-tests to compare difference in two proportions at 5% level of significance, and used bar graphs and Sankey diagrams to visualise the data. We analysed quantitative data using Stata, version 15.0 (StataCorp LLC, College Station, TX, USA).

For qualitative data, we prepared an *apriori* code list in ATLAS.ti, version 7.5 (ATLAS.ti, Berlin, Germany), after which we performed a thematic content analysis. The analysis process involved developing inductive codes based on the emerging key themes and sub-themes found within the data. The validity of the coding categories was measured via intercoder reliability. We extracted quotes from the raw data to provide evidence and present the respondents' opinions on each theme.

### Ethical clearance and consideration of ethical aspects

The Institutional Review Board of the icddr,b approved this study protocol. We obtained informed written consent from each study participant.

## RESULTS

### Antenatal care visits by place

Over 90% of the women in both urban and rural settings had at least one ANC visit at a health facility (urban: 94.0%, n = 4405; rural: 96.5%, n = 3119; *P* < 0.05), yet less than half of the women had at least four ANCs (urban: 42.1%, n = 4405; rural: 33.5%, n = 3119; *P* < 0.05) (**Table 1**). Though more women in the rural area had at least one ANC at a health facility, we observed a inverse situation for at least four ANCs.

**Table 1:** Distribution of types of facilities used for antenatal care visits by women in urban and rural areas

	n (%)
Sub-types of facilities where urban women visited (n = 4405)*	
Public (n = 1612, 36.6%)	
*MFSTC*†	1227 (76.1)
*Medical college hospitals*‡	135 (8.4)
*Upazila health complexes*§	102 (6.3)
*Other maternity hospitals*‖	97 (6.0)
*Community clinics*§	51 (3.2)
Private (n = 1098, 24.9%)	
*Chambers of qualified doctors*	536 (48.8)
*Private hospitals/clinics*	460 (41.9)
*Private medical college hospitals*	102 (9.3)
NGO (n = 1695, 38.4%)	
*Urban Primary Healthcare Service Delivery Project*	767 (45.3)
*BRAC clinics*	669 (39.5)
*Shurjer Hashi clinics*	106 (6.2)
*Marie Stopes’ clinics*	51 (3.0)
*Other NGOs*	102 (6.0)
**Sub-types of facilities where rural women visited (n = 3119)**†	
Public (n = 693, 22.2%)	
*Upazila health complexes*	244 (35.2)
*Upazila health & family welfare centres*	149 (21.5)
*Community clinics*	141 (20.3)
*District hospitals*	118 (17.0)
*Union sub-centres*	21 (3.1)
*Medical college hospitals*	10 (1.4)
*Mother and child welfare centres*	7 (1.1)
*Satellite clinic/EPI outreach*	3 (0.4)
Private (n = 2426, 77.8%)	
*Chambers of qualified doctors*	1009 (41.6)
*Private hospitals/clinics*	918 (37.8)
*Chambers of sub-assistant community medical office*	213 (8.8)
*Chambers of village doctors*	196 (8.1)
*Chambers of homeopathy doctors*	52 (2.2)
*NGOs*	35 (1.4)
*Pharmacies*	3 (0.1)

EPI – Expanded program on immunization, MFSTC – Mohammadpur Fertility Services and Training Centre, NGO – non-profit organisation, UH&FWC – *Upazila* health & family welfare centres, UPHSDP – Urban Primary Health Service Delivery Project

*Z-test for the difference in two proportions in urban area: public vs private: 36.6% vs 24.9%, *P* < 0.05; NGO vs private: 38.4% vs 24.9%, *P* < 0.05; MFSTC vs other public: 76.1% vs 23.9%, *P* < 0.05; UPHSDP vs other NGOs (Shurjer Hashi clinics, Marie Stopes’ clinics, other NGOs): 45.3% vs 15.2%, *P* < 0.05; BRAC vs other NGOs (Shurjer Hashi clinics, Marie Stopes’ clinics, other NGOs): 39.5% vs 15.2%, *P* < 0.05.

†MFSTC is a 100-bed mother and child hospital that provides family planning, maternal and child health, emergency obstetric care, and diagnostic services.

‡Shaheed Suhrawardy Medical College Hospital and Dhaka Medical College Hospital – tertiary health facilities.

§Women who migrated to the rural area during pregnancy received care from these facilities.

‖Mother and Child Health Training Institutes, two such facilities, provide maternal and child health, emergency obstetric care, and diagnostic services.

¶Z-test for the difference in two proportions in rural area: public vs private: 22.2% vs 77.8%, *P* < 0.05; *Upazila* health complexes vs other public: 35.2% vs 64.8%, *P* < 0.05; below *Upazila* health complex level (UH&FWCs, community clinics, union sub-centres, satellite clinics/EPI outreach) vs *Upazila* health complexes and above level (*Upazila *health complexes, mother and child welfare centres, district hospitals, medical college hospitals) public facilities: 45.3% vs 54.7%, *P* < 0.05; chambers of qualified doctor and private hospitals/clinics vs other privates: 79.4% vs 20.6%, *P* < 0.05.

When we examined the place of seeking ANC by type of facility, we found that about one-third of the total 4405 ANC visits in the urban area were made in public (36.6%, n = 1612) and NGO (38.4%, n = 1695) facilities each, while a quarter was made in private facilities (24.9%, n = 1098) (**Table 1**). In the urban area, the preference for either public (*P* < 0.05) or NGO facilities (*P* < 0.05) for ANC was significantly higher than for private facilities. However, most of the total 3119 ANC visits in rural areas (77.8%, n = 2426) were made in private facilities, while only about a quarter (22.2%, n = 693) were made in public facilities (*P* < 0.05).

A further analysis on types of facilities chosen for ANC in the urban setting revealed that three-fourths (76.1%, n = 1227) of the 1612 ANC visits in public facilities took place at Mohammadpur Fertility Services and Training Centre (MFSTC), a 100-bedded facility dedicated to maternity care services, and only 14.4% (n = 232) took place at other maternity or medical college hospitals (**Table 1**). A certain proportion of ANCs was sought at UHCs (6.3%, n = 1,02) and community clinics (CCs) (3.2%, n = 51) by those women who migrated to the rural area during pregnancy.

Of the 1695 ANC visits to NGOs, most were conducted at the Urban Primary Healthcare Services Delivery Project (UPHSDP), a government programme for the urban poor that is implemented through contracted-out NGOs (45.3%, n = 767), and BRAC, a large national NGO (39.5%, n = 669) (**Table 1**). The share of other NGOs (15.2%, n = 259) was much lower than the level of use of either the UPHSDP (*P* < 0.05) or BRAC (*P* < 0.05).

A total of 2426 ANC visits in the rural area took place in the private sector. These primarily included qualified doctors’ chamber (41.6%, n = 1009) and private hospitals/clinics (37.8%, n = 918) (**Table 1**). About one in ten (10.2%, n = 248) ANC visits were sought from other community-based skilled care providers, while the rest were sought from village doctors (8.1%, n = 196), homeopath doctors (2.2%, n = 52), and pharmacies (0.1%, n = 3).

One-third (35.2%, n = 244) of 693 ANC visits to public facilities in the rural area took place in the UHC, the main public facility in the sub-district level, while about one-fifth (19.5%, n = 135) was in above sub-district level facilities (**Table 1**). The use of below UHC level (Union Health & Family Welfare Centre (UH&FWC), CC, union sub-centres (USCs), satellite clinics, and Expanded Program on Immunization (EPI) outreach) public facilities for ANC by rural women was lower than that in UHC and higher level of facilities (45.3% vs 54.7%; *P* < 0.05).

### Pattern of switching facilities for antenatal care by trimester of pregnancy

**Figure 2:**
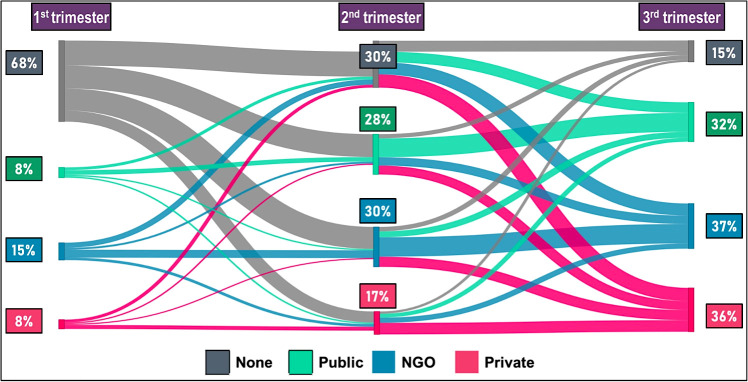
Women’s’ pattern of switching facilities for antenatal care in the urban area.

Overall, 68% of the women did not seek any ANC in the first trimester; 8% received ANC from both public and private facilities; and 15% from an NGO. In the second trimester, the use of ANC in the public sector increased from 8% to 28% (3.5-fold increase). The corresponding increases in the private and the NGO sectors were from 15% to 30% (2-fold increase) and from 8% to 17% (2-fold increase), respectively. When we examined the inflows and outflows of the clients in the second trimester of pregnancy, we observed a major inflow in the public sector from the group that did not receive ANC in the first trimester, and a relatively smaller outflow of clients towards the NGO and private sectors. We found a similar pattern at NGOs in the second trimester. However, for the private sector, we observed a major inflow from the group that received no ANC in the first trimester, with a relatively smaller outflow towards the public and NGO sectors. In the third trimester, we found a major inflow of the women in the private sector from rest of the three groups, bringing the public, NGO, and private sectors to have more or less equal share of ANC provision (**Figure 2**).

**Figure 3:**
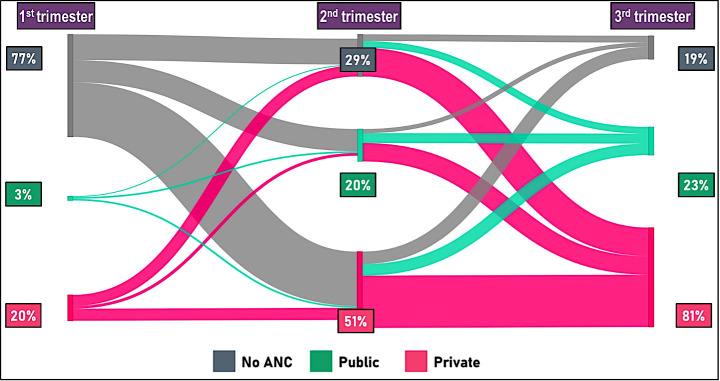
Women’s pattern of switching facilities for antenatal care in the rural area.

As there was only one NGO with a negligible stake in the rural area, its share was combined with that of the private sector (**Figure 3**). In this area, three-fourths (77%) of the participants did not seek any ANC in the first trimester; 3% received ANC from public facilities; and 20% from private facilities. In the second trimester, the use of public-sector facilities increased to 20%, and that for private-sector facilities increased to 51%. In both the sectors, the major contribution to this increase was from the group that received no ANC in the first trimester. In the third trimester, the private sector occupied the largest share, for which we observed a major inflow from the group that received no ANC in the second trimester, as well as from a large group from the public sector.

### Pattern of retention with health facilities for antenatal care

**Figure 4:**
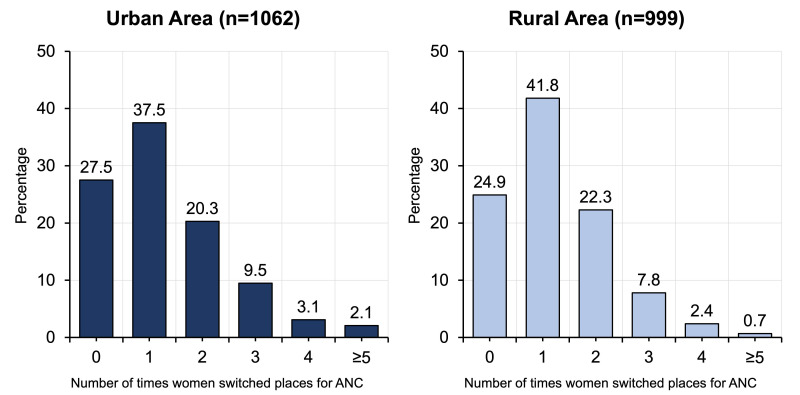
Number of times women (who had at least two antenatal care visits) switching facilities for ANC in the urban and rural areas.

Among the women who had at least two ANC visits, approximately a quarter (urban: 27.5%, n = 292; rural: 24.9%, n = 249; *P* > 0.05) stayed with the same facility for ANC (**Figure 4**). However, women in urban settings had a lower tendency of changing the facility one to two times compared to those in rural area (urban: 57.8%, n = 614; rural: 64.1%, n = 641; *P* < 0.05). Conversely, they changed ANC facilities three or more times less often their rural counterparts (urban: 14.7%, n = 156; rural: 10.9%, n = 109; *P* < 0.05).

Further investigation of the 292 urban women who did not switch their ANC facility showed that two large groups stayed with public facilities (45.2%, n = 132) and NGO facilities (37.0%, n = 108), while a relatively small group (17.8%, n = 52) stayed with private facilities (**Table 2**). Of the 132 women who remained in the public sector, most stayed with MFSTC (84.1%, n = 111). Concerning the NGO sector (n = 108), the majority remained with BRAC’s static clinics (59.3%, n = 64), followed by the UPHSDP (33.3%, n = 36).

**Table 2:** Distribution of facilities women did not change for antenatal care among those who had at least two antenatal care visits in the urban and rural areas

	n (%)
Sub-types of facilities where urban women visited (n = 292)*	
Public (n = 132, 45.2%)	
*MFSCT*‡	111 (84.1)
*Other maternity hospitals*§	11 (8.3)
*Medical college hospitals‖*	10 (7.6)
Private (n = 52, 17.8%)	
*Private hospitals/clinics*	25 (48.1)
*Chambers of qualified doctors*	21 (40.4)
*Private medical college hospitals*	6 (11.5)
NGOs (n = 108, 37.0%)	
*BRAC Clinics*	64 (59.3)
*Urban Primary Healthcare Service Delivery Project*	36 (33.3)
*Shurjer Hashi clinics*	8 (7.4)
**Sub-types of facilities where rural women visited (n = 249)**†	
Public (n = 22, 8.8%)	
*Upazila health complexes*	12 (54.5)
*District hospitals*	10 (45.5)
Private (n = 227, 91.2%)	
*Private hospitals/clinics*	106 (46.7)
*Qualified doctor’s chambers*	96 (42.3)
*Nurses/sub-assistant community medical officers*	11 (4.9)
*Village doctors*	8 (3.5)
*Private medical college hospitals*	6 (2.6)

MFSCT – Mohammadpur Fertility Services and Training Centre, NGO – non-profit organisation

*Z-test for the difference in two proportions in urban area: public vs private: 45.2% vs 17.8%, *P* < 0.05; NGO vs private: 37.0% vs 17.8%, *P* < 0.05; MFSCT vs other public: 84.1% vs 23.9%, *P* < 0.05.

†Z-test for the difference in two proportions in rural area: public vs private: 8.8% vs 91.2%, *P* < 0.05.

‡MFSCT is a 100-bed mother and child hospital that provides family planning, maternal and child health, emergency obstetric care, and diagnostic services.

§Mother and Child Health Training Institutes, two such facilities, provide maternal and child health, emergency obstetric care, and diagnostic services.

‖Shaheed Suhrawardy Medical College Hospital and Dhaka Medical College Hospital – tertiary health facilities.

However, we found a different scenario in the rural area. Among the 249 women who did not switch their ANC facility, almost all (91.2%, n = 227) stayed within the private sector, while only 8.8% (n = 22) stayed with the public sector (**Table 2**). Of 227 women who stayed with the private sector, most remained with private hospitals/clinics (46.7%, n = 106) and chambers of qualified private doctors (42.3%, n = 96).

### Factor influencing decision-making in choosing health facilities for antenatal care

Using the qualitative approach, we further explored the factors influencing the women’s decision to choose certain types of facilities for ANC. In the urban setting, large, dedicated public maternity hospitals that had one-stop service provision at a reasonable cost attracted women and their families for ANC. One woman attending a public maternity hospital stated:

*This hospital has all the facilities for pregnant mothers, from ANC to delivery. Also, the doctors are well-behaved; so [are] the nurses and the janitors.*

In addition, the high concentration of NGO facilities backed up by community health workers in the resource-poor urban setting motivated the pregnant women to receive ANC from the affiliated NGOs of the respective community health workers. Both public and NGO facilities had specialist doctors to provide ANC and other maternal and child health care services, which influenced the women’s decision to choose these facilities. Furthermore, low-cost and discounted services from both public and NGO facilities also worked as a pull factor. One urban woman who was informed by her neighbour about the NGOs’ discounted services stated:

A staff member from an NGO hospital lives next door to me. We are low-income people; so she arranged for me a red card at that hospital. If you have a red card, you don't have to pay for health care services in that hospital; you only have to buy medicines. Everything else is available free of charge. I got registered with the red card during my seven months of pregnancy. Since then, I have been going to that NGO hospital.

*Factors influencing the rural women and their family members to choose a private facility for ANC included a high concentration of private facilities at the sub-district and district levels being supported by strong marketing strategies linked with the village doctors and health workers in the community, as well as brokers at public facilities sending patients to the private facilities in exchange for financial benefits.*

Also, the private facilities maintained a network with local specialist care providers from the public sector who attended the private facilities on an on-call basis. Some of the private facilities also invited reputed specialists from other districts mainly from Dhaka to visit patients at their facilities on the weekends to fulfil the strong demand from the women and their families for specialist care providers. One rural pregnant woman who went to a private clinic for ANC said:

*I had chest pain when I was pregnant. My parents suggested me to visit the Upazila-level public hospital. Accordingly, I visited that hospital but I did not find a doctor on that day. Then, I returned home and talked to a village doctor about my chest pain. The village doctor suggested me to visit a gynecologist who is available in a private clinic of the district town.*

However, the sub-district-level public facilities consistently suffered from lack of readiness due to unavailability of specialist care providers, shortage of technical and other support staff, and the unavailability of the required diagnostic tests (including ultrasonography (USG) services) for which the women had a strong demand. These shortcomings of public facilities often compelled the women and their family members to go to private facilities. One rural woman who went to a private facility for diagnostic services stated:

*The doctor at the district hospital (public) informed us that they will be able to perform some tests in the hospital but I have to do the USG from outside of the hospital to get the report immediately. There are lots of patients in the district hospital, and it might take two days to receive the USG report from there. For that reason, I went to a private clinic to have my USG and other tests.*

**Figure 5:**
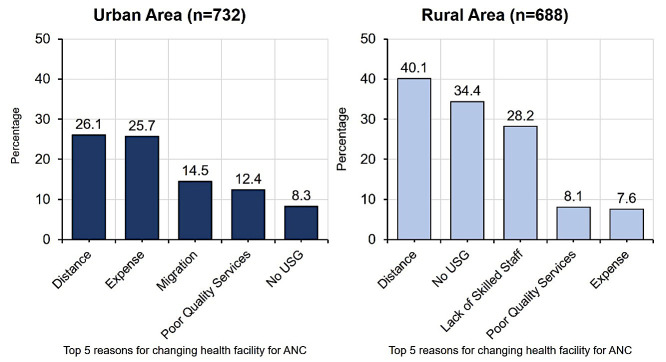
Top five reasons for changing facilities for antenatal care.

Half of the women in the urban area mentioned distance (26.1%) and expenses (25.7%) as the main factors among the top five reasons for changing their ANC facility. Migration (14.5%) was the third most reported reason, as a group of women had moved to their village homes before delivery. In the rural area, after distance (40.1%), no USG facilities (34.4%) and lack of skilled staff (28.2%) were the main reasons for changing the ANC facilities. Poor service quality was one of the five reported reasons in both settings (urban: 12.4%; rural: 8.1%) (**Figure 5**). The qualitative investigation exploring the reasons behind changing ANC facility also supported the quantitative findings and highlighted other factors as the reasons for changing facilities in both urban and rural areas, such as no provision for USG; high cost of services; poor-quality care; no expert provider; excessive distance; and poor road infrastructure. One rural woman who changed her ANC facility due to distance and poor road communication stated:

*The facility is far from our house; also, the road is full of holes. I have no one to accompany me to the health facility … If the facility is close to my house, then anyone of my family can come to see me anytime. This is why I have changed the facility.*

### Usage of ultrasonography by the pregnant women during antenatal period

**Figure 6:**
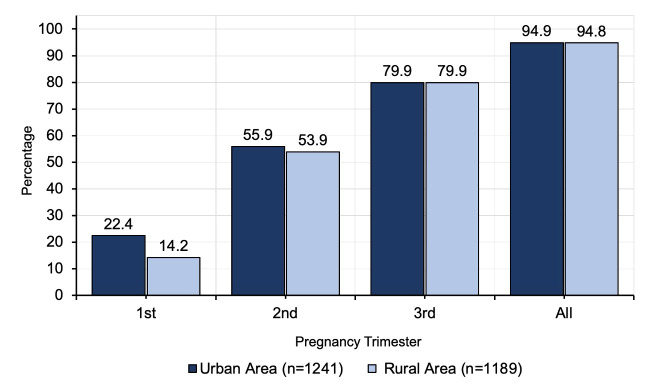
Usage of ultrasonography at least once by the pregnant women during antenatal care by trimester.

We observed an increasing trend of having USG over the pregnancy period in both the settings (**Figure 6**). In the first trimester, significantly more women in the urban area had USG than their rural counterpart (urban: 22.4%; rural: 14.2%; *P* < 0.05). However, this difference diminished with the progression of the trimester, with 79.9% of women in the third trimester from each urban and rural areas having at least one USG. Regardless of the trimester, around 95% of the women from both study areas had at least one USG at any point of their pregnancy period.

## DISCUSSION

Our findings showed the decision-making process women undergo in receiving ANC from different types of facilities in a rural and an urban area of Bangladesh. Although many women from both urban (94.0%) and rural areas (96.5%) made at least one ANC visit to facilities during their entire pregnancy period, the proportions of those who made four or more ANC visits (urban: 42.1%: rural: 33.5%) were inadequate in both sites. In the urban area, pregnant women primarily visited NGOs (38.4%) and public facilities (36.6%) for ANC, followed by private facilities (24.9%). In the rural area, private facilities were visited more often (77.8%). The reputation of the facility; the availability of skilled care providers and diagnostic tests; and the provision of USG services under one roof were important factors influencing the pregnant women’s choice of facility for ANC. Both urban and rural pregnant women in our study preferred receiving USG, as most had at least two USG tests during their pregnancy period. During the first trimester of pregnancy, most of the urban and rural women in the current study did not tend to receive any ANC, which was consistent with the recent research findings by Islam et al. [26].

Our findings on the high use of at least one ANC (urban: 94.0%; rural: 96.5%) and the low use of at least four ANCs (urban: 42.1%; rural: 33.5%) somewhat align with those from the rural area within the BDHS 2022 [15] (urban: one ANC 86%, four ANCs 56.9%; rural: one ANC 92%, four ANCs 34.2%). However, in urban area, there was a discrepancy between the BDHS 2022 and our findings that may be due to our selection of women from the poor urban setting, while BDHS collected representative sample from the urban population. Nevertheless, our findings of 42.1% having at least four ANCs are consistent with the findings of 42.0% having at least four ANCs reported by another study in urban slums of Dhaka in 2022 [27], while our findings on the low use of ANCs by the urban poor are supported by other studies [28,29], where they proved that the ‘urban advantage’ theory for using maternal health care services does not function for urban poor in developing countries.

The health care delivery system in Bangladesh relies primarily on public, non-profit NGOs and the for-profit private sector to provide health care services to the community. Based on the findings of a study conducted by Rezaeian et al. in Iran [23], we can hypothesise that the utilisation of the type of health care services by an individual is indicative of their socioeconomic background. In the poor urban area of our study, many women sought ANC from NGO facilities due to their lucrative offers, such as discounted health care services and provision of free medicine. Relatively high use of NGO-based services in our study is also explained by their community-oriented strategies and flexibility in project planning and implementation as have been proven in other settings [30,31].

Alongside the NGO facilities, two large public facilities located in the urban setting of our study were a specialised maternity hospital (MFSTC) and a medical college hospital [32,33]. According to the Bangladesh Health Facility Survey 2017, medical college hospitals have reputation for having specialised doctors and providing more comprehensive care [34]. Nevertheless, the poor pregnant women in the urban area chose the specialised maternity hospital (MFSTC) over the medical college hospital due to the availability of one-stop services [35]. Similar findings were observed in studies conducted in Iran and Malawi, indicating that maternity hospitals were advantageous for availability of specialist doctors, facilities for normal delivery, caesarean section, and good management, despite the crowdedness and lack of sufficient resources, which led to women seeking care there [35–37]. These factors not only drew women to facilities with one-stop services, but also helped them decide to return to there for pregnancy care [38].

However, we observed an inverse scenario in the rural area, where a higher proportion of pregnant women sought health care from private rather than public facilities. The scarcity of specialist health care providers, unavailability of diagnostic services, poor amenities, and unprofessional behaviour of the health care providers in public facilities have proven to be significant obstacles in rural health care service delivery [34,39]. In Bangladesh, the community-level health facilities of rural areas and hospitals at the sub-district and district levels are not equipped with proper supplies and resources [34,39,40]. Similar to our findings, other studies conducted in Bangladesh have shown that the private health facilities in rural areas use different approaches considering the area-specific context, such as providing female specialists; undertaking rigorous market promotion; engaging brokers and agents in the community and in hospitals; and providing diagnostic services [41,42].

In our study, around a quarter of women (urban: 27%; rural: 25%) visited the same facilities of their choice for ANC during pregnancy due to the availability of the required services in one place, regardless of the type of facility. Although we found no study during our literature review that examined the underlying causes of retention and attrition of pregnant women in the same facility, several studies in South Asia and Africa have explored the factors influencing the satisfaction of pregnant women while seeking ANC [43–47]. The behaviour of the health care staff, technical specialty of the care providers, waiting time, laboratory services, cleanliness, and medicine supply were some of the key factors for choosing facilities for ANC [43–47]. Passive interpretation of the above elements of care functions as a driving factor for pregnant women to change facilities for ANC. This evidence is supported by our findings on factors influencing retention (good behaviour of the health care staff, availability of USG, specialist care providers) and attrition of women (unavailability of health care providers, distance, waiting time) at the same facility. A recent systematic review conducted by Strong et al. [48] examined the role of private sectors in maternal health care service delivery in low- and middle-income countries and found that pregnant women from these contexts mainly preferred facilities where they found less waiting time, less crowd, good counselling, and good behaviour from the clinical staff, which align with our study findings.

### Strengths and limitations

The main strength of our research is its cohort study design with follow-up visits to the pregnant women over the course of pregnancy which allowed us to collect information on decision-making process for pregnancy care by minimising the recall bias. Another strength is the mixed method approach that helped us explore the complex decision-making process for care-seeking by the women and their family members.

The main limitation of our study is the use of different approaches in selecting the subjects from urban and rural settings. Specifically, we selected subjects in urban areas through house-to-house visit by identifying the poor households based on specific characteristics; in the rural area, we selected subjects based on the local surveillance system. However, we kept the criteria of enrolling the pregnant women identical in both settings (<27 weeks of gestation). The second limitation of our study is the possibility of the results being affected by the collection period coinciding with the coronavirus disease 2019 (COVID-19) pandemic in the urban area. The third limitation of our study is that we initiated data collection in two different time periods in urban and rural sites with only a one-month overlap.

## CONCLUSIONS

Our study showed that while seeking ANC, pregnant women and their families, regardless of their area of residence, prefer health care facilities that offer one-stop comprehensive services. In the context of urban environments, they prefer accessing large public maternity hospitals with one-stop services. Thus, the establishment of such hospitals with one-stop services in different zones of urban areas could facilitate the provision of comprehensive maternal health care services in a centralised manner. In rural environments, we found that an insufficient level of readiness in public facilities leads pregnant women and their families to seek ANC from private facilities. This necessitates the provision of appropriate diagnostic services (e.g. USG and laboratory tests) and necessary human resources and logistical support to the community- and sub-district-level public facilities and their referral facilities at the district level. In the poor urban setting, NGO facilities play a crucial role in addressing deficiencies in the provision of maternity care services by public sector; the provision of NGO services should thus be sustained and enhanced in the urban regions where adequate public services are lacking. This could be achieved through an innovative initiative of public-private partnership. In the rural setting, private health care facilities apply proactive marketing approach to attract clients. This aspect could be counteracted through the implementation of high-quality services in rural public facilities. Strong policy decision is needed to ensure the provision of one-stop quality maternity services in public and NGO sectors that will help mitigate the dominance of the private sector and, ultimately, benefit the poor in fulfilling their needs of maternity care services.
